# Identification and Morphogenesis of Vestibular Atrial Septal Defects

**DOI:** 10.3390/jcdd7030035

**Published:** 2020-09-10

**Authors:** Rohit S. Loomba, Justin T. Tretter, Timothy J. Mohun, Robert H. Anderson, Scott Kramer, Diane E. Spicer

**Affiliations:** 1 Division of Cardiology, Advocate Children’s Hospital, Chicago, IL 60453, USA; 2 Heart Institute, Cincinnati Children’s Hospital Medical Center, Cincinnati, OH 45229, USA; justin.tretter@cchmc.org; 3 Francis Crick Institute, London NW1 1AT, UK; tim.mohun@gmail.com; 4 Cardiovascular Research Centre, Institute of Genetic Medicine, Newcastle University, Newcastle upon Tyne NE1 3BZ, UK; sejjran@ucl.ac.uk; 5 Division of Pediatric Cardiology, University of Florida, Gainesville, FL 32611, USA; scott.kramer888@gmail.com (S.K.); spicerpath@hotmail.com (D.E.S.)

**Keywords:** vestibular atrial septal defect, atrial septal defect, cardiac development, atrial septum

## Abstract

*Background*: The vestibular atrial septal defect is an interatrial communication located in the antero-inferior portion of the atrial septum. Reflecting either inadequate muscularization of the vestibular spine and mesenchymal cap during development, or excessive apoptosis within the developing antero-inferior septal component, the vestibular defect represents an infrequently recognized true deficiency of the atrial septum. We reviewed necropsy specimens from three separate archives to establish the frequency of such vestibular defects and their associated cardiac findings, providing additional analysis from developing mouse hearts to illustrate their potential morphogenesis. *Materials and methods*: We analyzed the hearts in the Farouk S. Idriss Cardiac Registry at Ann and Robert H. Lurie Children’s Hospital in Chicago, IL, the Van Mierop Archive at the University of Florida in Gainesville, Florida, and the archive at Johns Hopkins All Children’s Heart Institute in St. Petersburg, Florida, identifying all those exhibiting a vestibular atrial septal defect, along with the associated intracardiac malformations. We then assessed potential mechanisms for the existence of such defects, based on the assessment of 450 datasets of developing mouse hearts prepared using the technique of episcopic microscopy. *Results*: We analyzed a total of 2100 specimens. Of these, 68 (3%) were found to have a vestibular atrial septal defect. Comparable defects were identified in 10 developing mouse embryos sacrificed at embryonic data 15.5, by which stage the antero-inferior component of the atrial septum is usually normally formed. *Conclusion*: The vestibular defect is a true septal defect located in the muscular antero-inferior rim of the oval fossa. Our retrospective review of autopsied hearts suggests that the defect may be more common than previously thought. Increased awareness of the location of the defect should optimize its future clinical identification. We suggest that the defect exists because of failure, during embryonic development, of union of the components that bind the leading edge of the primary atrial septum to the atrioventricular junctions, either because of inadequate muscularisation or excessive apoptosis.

## 1. Introduction

The vestibular defect is a true septal defect located in the antero-inferior muscular rim of the oval fossa [1]. Initially noted and described as a solitary finding, a formal investigation of its frequency, as far as we are aware, has yet to be completed using large series of autopsied hearts [2,3]. We describe here the findings from our review of necropsy specimens from three separate archives. We also provide evidence from examination of developing murine hearts, which offer an explanation for the morphogenesis of the lesion.

## 2. Methods

We analyzed all hearts in the Farouk S. Idriss Cardiac Registry at Ann and Robert H. Lurie Children’s Hospital in Chicago, IL, the Van Mierop Archive at the University of Florida in Gainesville, Florida, and the archive at Johns Hopkins All Children’s Heart Institute in St. Petersburg, Florida, paying particular attention to the integrity or otherwise of the antero-inferior margin of the oval fossa. In hearts where there had been surgical intervention, we also took note of the findings as described in the original autopsy reports so as to ensure that any unexpected communications observed were not the results of the prior surgery. The findings are summarized in Table 1. We then assessed the formation and closure of the atrial septum in mouse embryos as revealed using the technique of epicsopic microscopy, the mechanisms of septal development known to be comparable in mice and humans [1,4]. Parkes mice were used for these investigations. Over 400 datasets were examined, providing evidence of the temporal changes occurring during septation of the atrial chambers in the mouse heart. The datasets covered the period from the eleventh day of intrauterine development to term, which in the mouse usually occurs on the nineteenth day of development.

## 3. Results

### 3.1. Human Necroscopy Specimens

**Figure 1:**
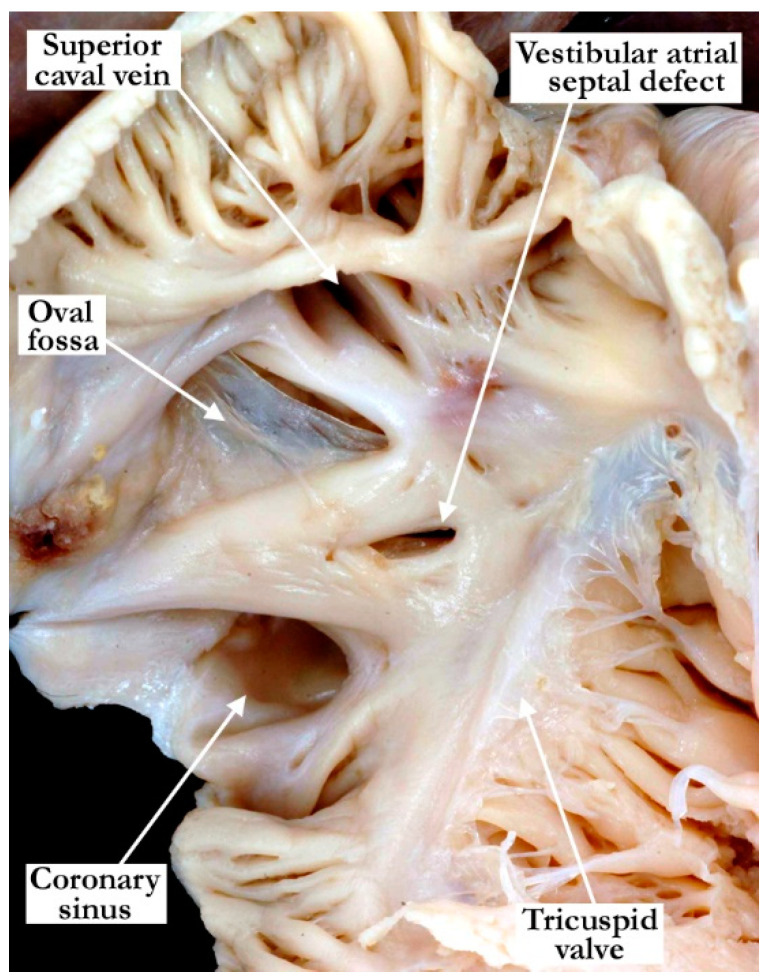
The vestibular atrial septal defect seen in this image is midway between the antero-inferior rim of the oval fossa and the hinge point of the tricuspid valve. This is the most common position for the defect. There is a dilated coronary sinus secondary to a persistent left superior caval vein.

**Figure 2:**
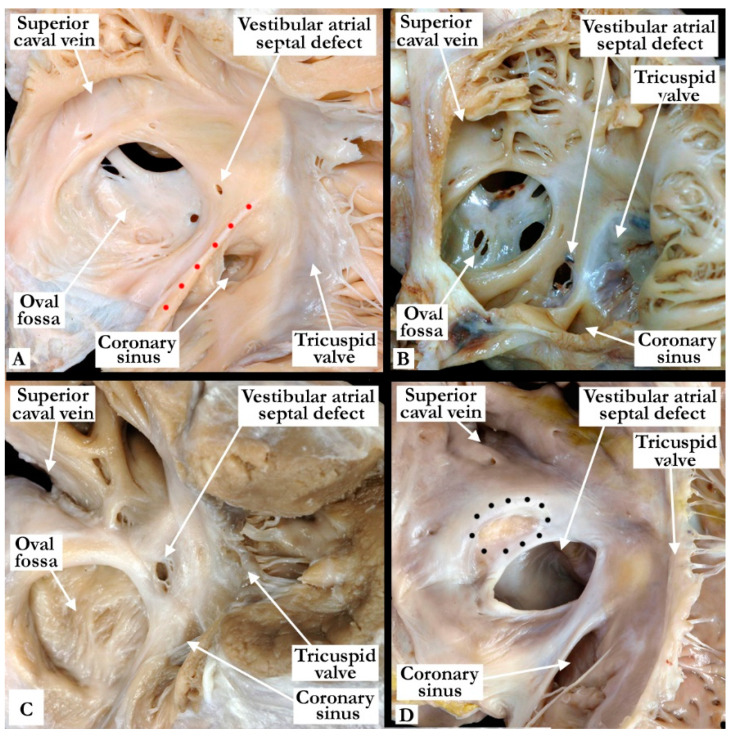
The images show the variations in size and position of vestibular atrial septal defects. In (Panel **A**), we show a small defect within the antero-inferior rim of the oval fossa. There is also a fenestrated flap valve at the floor of the oval fossa. The Eustachian valve (red dots) is prominent in this specimen. In (Panels **B**,**C**), the defects are larger and focally fenestrated with the defect shown in (Panel **B**) in a more inferior position, while the defect seen in (Panel **C**) is toward the most superior extent of the margins of the true atrial septum. There is an intact flap valve in the heart shown in (Panel **C**), and a fenestrated flap valve at the floor of the oval fossa in the heart seen in (Panel **B**). In (Panel **D**), we show an exceedingly large vestibular defect. This was an incidental finding at autopsy in a 46-year-old patient who died secondary to a stroke. It was approximately 2 cm in diameter. The borders of the oval fossa are marked with black dots and the foramen was closed.

**Figure 3:**
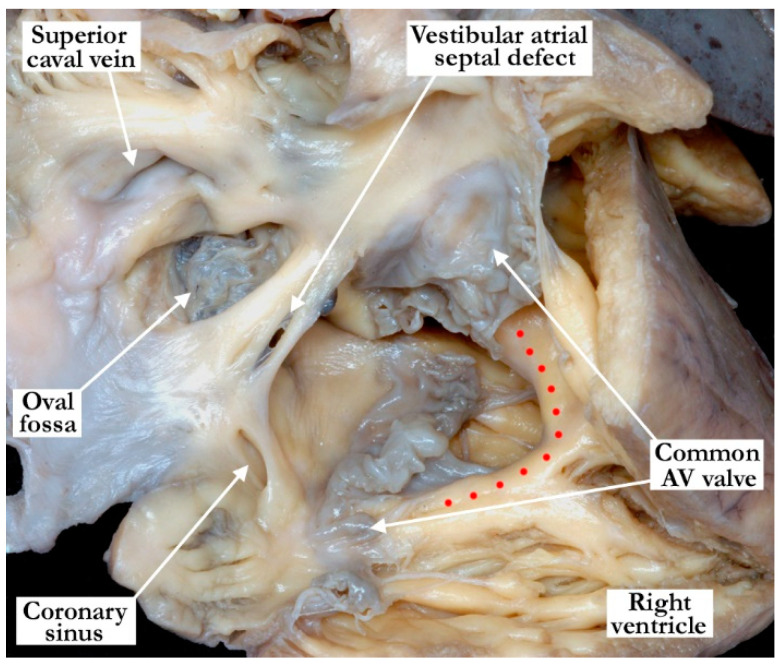
This heart with a common atrioventricular junction guarded by a common valve is viewed from the right ventricle, with the ventricular septum marked with red dots. There is a competent flap valve at the floor of the oval fossa, and a vestibular septal defect in the antero-inferior muscular rim, directly adjacent to the atrial component of the atrioventricular septal defect.

We analyzed a total of 2100 specimens, with 68 (3%) found to have a vestibular atrial septal defect. We use this denominator of 68 hearts with vestibular atrial septal defects for our subsequent descriptions. All the defects were found in the antero-inferior muscular rim of the oval fossa, typically in the area antero-superior to the mouth of the coronary sinus (Figure 1). This area, which provides the antero-inferior muscular buttress of the oval fossa, includes the narrow vestibule of the right atrium that lies between the antero-inferior rim and the hinge of the septal leaflet of the tricuspid valve. A slight variation was observed in the position of the defect, with its size also varying (Figure 2). Some of the defects were slit-like and tiny, but large defects were also found, as in one of the hearts examined from an adult patient (Figure 2). The defects opened to the left atrium adjacent to the hinges of the leaflets of the mitral valve, with retention of the normal off-setting of the hinges of the atrioventricular valvar leaflets in the hearts having separate atrioventricular junctions (Figure 3).

**Figure 4:**
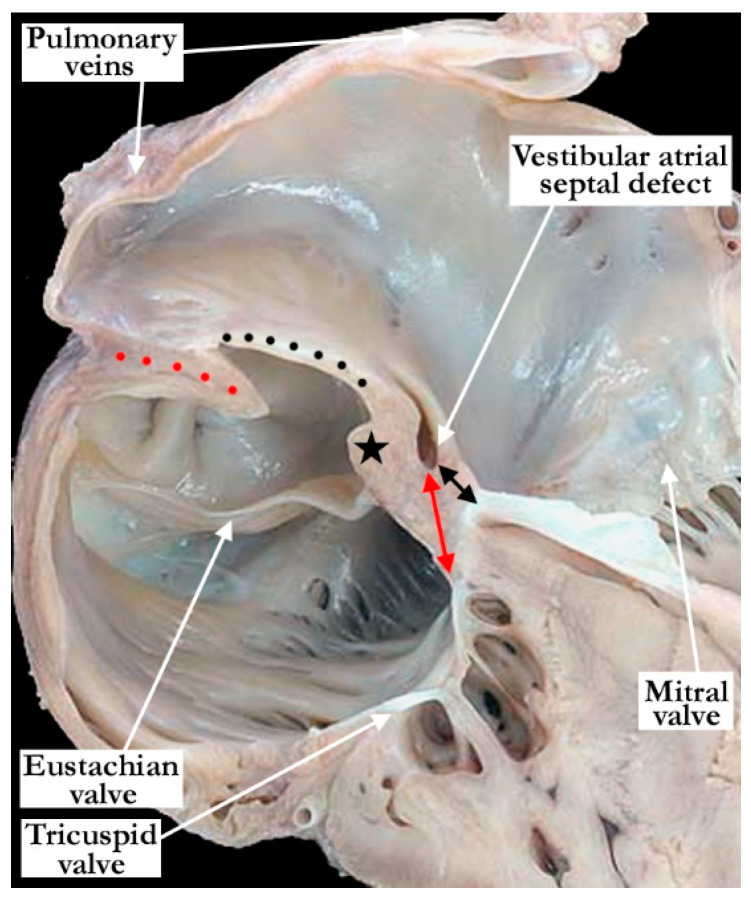
The heart is sectioned in a four-chamber plane to demonstrate the position of a vestibular atrial septal defect in the inferior muscular buttress. The defect occurs between the vestibules of the right and left atrium. When there is normal offsetting of the atrioventricular valves, as in the heart shown, the defect is typically closer to the mitral valve (double-headed black arrow) and farther from the tricuspid valve (double-headed red arrow). The antero-inferior rim of the oval fossa is marked with a black star. In this image, the opening of the defect to the right atrium is not seen. The flap valve (black dots) at the floor of the oval fossa was well-formed and overlaps the superior interatrial fold (red dots). Note the imperforate tricuspid valve in this heart which also had pulmonary atresia with an intact ventricular septum.

In terms of associated lesions, all hearts had a usual atrial arrangement, with right-sided superior caval veins that drained into the roof of the right-sided atrium. A left-sided superior caval vein was present in four (6%) of the hearts, in all instances draining into the coronary sinus. The pulmonary veins returned to the left atrium in a normal fashion in all of the specimens. The morphologically right atrium was dilated in 42 hearts (62%), with the morphologically left atrium being dilated in 20 (29%), and hypoplastic in two (3%). An additional defect was present in the oval fossa in 53 hearts (78%), with an additional coronary sinus interatrial communication found in three (4%). A common atrioventricular junction was present in 10 (15%), accompanied by the anticipated “ostium primum” atrial component of the associated atrioventricular septal defect. In these hearts, the atrial septum itself was well-formed, with a recognizable oval fossa. The vestibular defect itself was in the leading edge of the septum, adjacent to the “ostium primum” defect (Figure 4). The atrioventricular connections were concordant in 65 (94%), discordant in one (2%), solitary and right-sided in one (2%), and solitary and left-sided in one (2%). An abnormality of the right-sided atrioventricular valve was noted in 40 hearts (59%), with a left-sided atrioventricular valvar abnormality noted in 19 (28%). Abnormalities of the atrioventricular valves included both stenosis and dysplasia. Ventricular topology was right-handed in all the hearts except for the one with discordant atrioventricular connections (98%). The ventriculo-arterial connections were concordant in 60 (89%), discordant in five (7%), and double outlet right ventricle in three (4%). Pulmonary atresia was present in 17 hearts (25%), while aortic atresia was present in one (2%). The aorta was most commonly posterior and rightward (58, 84%). Right ventricular outflow tract obstruction was present in 37, (58%) most commonly at the level of the arterial valve. An interventricular communication was present in 31 (46%). The aortic arch coursed over the left-sided pulmonary artery and bronchus in 66 (98%). Coarctation of the aorta was present in four (6%), with interruption of the aortic arch present in two (3%). The coronary arterial origins were abnormal in three (6%). An arterial duct was present in 33 (49%), ligated in eight (11%), and absent in 27 (40%). The cardiac apex was leftward in all but one heart, which had an apex that was pointed towards the midline. A history of trisomy 21 was documented in five patients (7%), with a history of 22 q deletion documented in one (2%).

### 3.2. Developmental Considerations

Since the defects observed in the autopsied hearts were all found either within the antero-inferior buttress of the oval fossa, or at the site of anchorage of the floor of the fossa to the buttress, we reasoned that knowledge of normal development of the atrial septum might permit us better to understand the morphology and location of the defects. We studied, therefore, the temporal changes occurring during septation of the atrial chambers in the mouse heart, taking advantage of our access to over 400 datasets prepared from developing mouse embryos which cover the period from the eleventh day of intrauterine development to term, which in the mouse usually occurs on the nineteenth day of development. The key stages take place during embryonic day (E) 10.5, with the initial appearance of the primary atrial septum, and continue through E13.5, when it is possible to recognise the margins of the oval fossa, along with the formation of the oval foramen (foramen ovale). Persistent patency of the oval foramen, of course, continues until term. The foramen, nonetheless, is capable of being closed from E13.5 until term in the developing murine heart. The floor of the fossa, derived from the primary atrial septum, is itself usually intact from E13.5 onwards to term. The period from E10.5 through E13.5 corresponds to the sixth through the eighth week of human intrauterine development.

**Figure 5:**
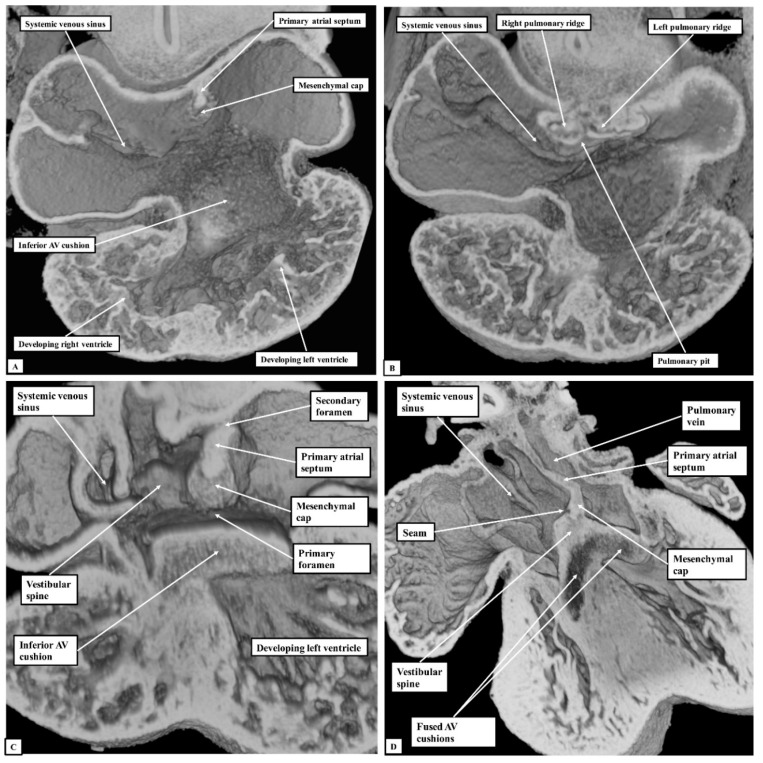
The images show ventral (Panel **A**) and more dorsal (Panel **B**) sections taken from the same episcopic dataset prepared from a developing mouse embryo sacrificed on the eleventh day of intrauterine development (E10.5). The lower panels show similar four-chamber sections at the stages of embryonic days 11.5 (Panel **C**) and 13.5 (Panel **D**) Abbreviation: AV—Atrioventricular.

At E10.5, the primary atrial septum can be recognised as a muscular ridge growing from the roof of the atrial component of the heart tube, which is a common structure at this stage of development. By this stage, the borders of the confluence of the systemic venous tributaries with the common atrial cavity are already marked by the venous valves, with the opening of the systemic venous sinus already committed to the right side of the developing atrial chamber (Figure 5A). The atrioventricular canal at this early stage is committed exclusively to the cavity of the developing left ventricle, although the parietal wall of the right atrium is already in continuity with the wall of the developing right ventricle in the roof of the embryonic interventricular communication. The primary septum itself carries a mesenchymal cap on its leading edge, with the space between the cap and the atrioventricular cushions representing the primary atrial foramen. Dorsally at this stage, the atrial walls are continuous with the pharyngeal mesenchyme through the persisting mesocardial connection. Growth from the mesenchyme into the right margin of the mesocardium has already produced inequality in size of its rims, which now protrude into the atrial cavity as the pulmonary ridges (Figure 5B). As yet, there is no formation of the pulmonary veins, but the floor of the dorsal mesocardial connection, known as the pulmonary pit, will eventually provide the site of connection between the developing pulmonary veins and the atrial cavity.

By the next day of intrauterine development (E11.5—Figure 5C), the primary atrial septum has grown towards the cushions developed within the atrioventricular canal. The canal itself has now expanded rightwards so as to bring the cavity of the right atrium into direct connection with that of the right ventricle. The upper margin of the primary foramen has now broken away from the atrial roof to form the secondary atrial foramen, while the size of the primary foramen is much reduced. By now, the growth of mesenchymal tissue into the rightward margin of the dorsal mesocardial connection has produced an intraluminal swelling that overlaps the rightward and dorsal extent of the leading edge of the primary atrial septum. This is the vestibular spine, also known as the dorsal mesenchymal protrusion. The pulmonary vein by this stage has canalised within the pharyngeal mesenchyme, opening to the atrium through the pulmonary pit. The growth of the vestibular spine ensures that the pulmonary venous orifice is committed to the developing left atrium. By E13.5, the mesenchymal cap on the atrial septum has fused with the atrial margins of the atrioventricular cushions, which themselves have fused together to separate the atrioventricular canal into the developing tricuspid and mitral valvar orifices (Figure 5D—Lower right-hand panel). The rightward margin of the cap is now itself overlapped by the vestibular spine, with an obvious seam noted between these structures. The two entities together form the caudal rim of the developing oval fossa. Both by now are losing their mesenchymal characteristics and are attaining the same texture of the walls of the cardiac chambers, which we interpret as indicating their muscularisation. The cranial margin of the fossa can now be recognised as a small ridge formed to the right side of the cranial attachment of the primary atrial septum.

**Figure 6:**
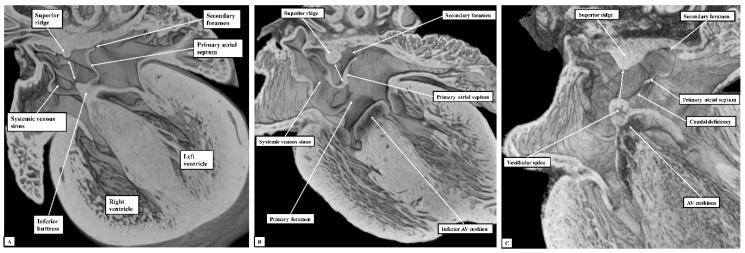
The images show sections prepared in the four-chamber plane from mouse embryos sacrificed at the sixteenth day of development (E15.5). (Panel **A**) shows the normal arrangement, with the primary atrial septum forming the floor of the oval fossa (double-headed white arrow). (Panel **B**) shows an “ostium primum” defect, with common atrioventricular junction due to failure of formation of the vestibular spine. (Panel **C**) shows a large defect in the primary septum adjacent to the vestibular spine, thus producing a caudal defect within the oval fossa. The double-headed white arrow shows the margins of the oval fossa. AV—Atrioventricular.

**Figure 7:**
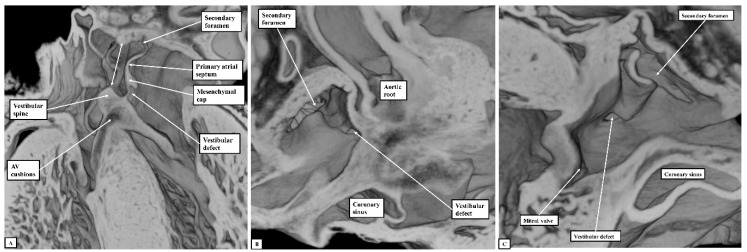
The images show the features of the vestibular defect, as was found in four of the datasets. The dataset used to prepare the figure is sectioned so as to show the appearance of the defect in the four-chamber plane (Panel **A**), and as seen from the right (Panel **B**) and left (Panel **C**) sides. The double-headed white arrow in the left-hand panel shows the margins of the oval fossa. AV—Atrioventricular.

If development proceeds in a normal fashion, the margins of the oval fossa are intact by E15.5. We examined episcopic datasets prepared from 48 mouse embryos at this stage. In 38 of the mice, the findings show the normal arrangement, with the primary septum folded on itself cranially, its length being considerably greater than the length of the fossa (Figure 6A). In these 38 datasets, the vestibular spine and mesenchymal cap fused together to form the caudal rim of the fossa, with obliteration of the seam initially seen between the two components during E13.5 (Figure 5D). The primary septum now forms the floor of the oval fossa, with the space between its cranial margin and the atrial roof forming the oval foramen, or secondary atrial foramen. The excessive length of the septum relative to the dimensions of the fossa provides the mechanism that permits closure of the foramen subsequent to birth. In 10 of the datasets prepared from mice sacrificed at E15.5, however, we found deficiencies of either the inferior rim of the oval fossa, or the attachment of the leading edge of the primary septum. In two, the findings could be attributed to the failure of formation of the vestibular spine (Figure 6B), with additional failure of growth of the primary septum in one of the two. In both, the lack of growth of the spine was associated with persistence of a common atrioventricular junction, with the atrioventricular cushions fused to each other, and also to the crest of the muscular ventricular septum, in other words producing an “ostium primum” defect. In four further datasets, although the vestibular spine had protruded in anticipated fashion to form the caudal margin of the oval fossa, thus separating the right and left atrioventricular junctions and forming the caudal rim of the fossa, the primary septum itself was deficient adjacent to the caudal rim, thus producing a hole in the floor of the fossa adjacent to its antero-inferior buttress, in other words, an “ostium secundum” defect (Figure 6C) The defect was large in two of the datasets but smaller in the remaining two. In the remaining four datasets, the mesenchymal cap had failed to fuse with the vestibular spine. The spaces in the floor of the oval fossa in these four hearts (Figure 7), therefore, are directly comparable to the vestibular defects found in our autopsied hearts.

## 4. Discussion

The majority of interatrial communications can be divided anatomically into two groups: those located within the rims of the oval fossa, and those located outside the oval fossa [5]. The latter lesions include the sinus venosus, coronary sinus, and “ostium primum” defects. The true defects of the atrial septum are those confined within the rims of the oval fossa. Additionally known as “ostium secundum” defects, they exist because of deficiencies of the floor of the fossa, which is derived from the primary atrial septum [1]. Some time ago, another form of true atrial septal defect was described by Sharratt and colleagues. It was located within the anterior-inferior rim of the fossa [3]. This defect, called a vestibular defect, produced a communication between the vestibules of the mitral and tricuspid valves. As we now show, such defects can also be found in the setting of a common atrioventricular junction.

These defects are a result of either inadequate muscularization of the vestibular spine and mesenchymal cap during development, or excessive apoptosis within the developing antero-inferior septal component, the vestibular defect represents an infrequently recognized true deficiency of the atrial septum. The detailed morphogenesis of the atrial septum is described at length in the results.

The initial account of such a vestibular defect was followed by a description by Fukuda and colleagues following surgical correction [2]. As we now confirm, this part of the atrial septum is produced by the fusion of the vestibular spine, or dorsal mesenchymal protrusion, with the mesenchymal cap carried on the leading edge of the primary atrial septum. Webb and colleagues had emphasised the significance of the vestibular spine in completing atrial septation, although the spine itself was first described by His in the 19th century [6]. The importance of the spine was then endorsed by Blom and colleagues, who described the process of atrioventricular septation as requiring not only the fusion of the endocardial AV cushions and downward growth of the muscular primary atrial septum, but also the ventral proliferation of the spine [7]. The latter investigators found a reduced amount of extracardiac mesenchyme in the vestibular spine of human embryos with Down’s syndrome, leading to a lack of ventral protrusion of this mesenchymal mass in the setting of a common atrioventricular junction. The role of the spine was then reinforced by Snarr and colleagues, who chose to describe it as the dorsal mesenchymal protrusion. They demonstrated, in an elegant fashion, its relationship to the newly discovered second heart field [8]. As we have now shown using episcopic microscopy, this non-endocardially derived mesenchymal component, along with the mesenchymal cap carried on the leading edge of the primary atrial septum, eventually fuse to form the definitive antero-inferior buttress of the oval fossa. As we also show, it is improper fusion of the spine with the remaining components of the developing septum that is the essence of the vestibular defect.

All of the defects that we found are comparable to the lesion initially reported by Sharratt and colleagues [3]. Recognition of the presence of the vestibular defect is important since percutaneous closure of isolated or multifenestrated defects within the fossa could still leave residual shunting through the vestibular defect [9,10]. Our retrospective review of autopsied hearts now suggests that the defect may be more common than previously thought. It is also perhaps pertinent that none of the original autopsy reports prepared for the hearts we investigated described the presence of the vestibular defects. It could well be, therefore, that the defects could go unnoticed during autopsy examination unless specifically sought. It is also possible that the right atrial entrance to the defect could be mistaken for a Thebesian vein. In all of our specimens, we excluded this possibility by probing the defect, confirming that it represented a true interatrial communication. Diagnosis also represents an echocardiographic challenge. When the vestibular defect is present concomitant with an oval fossa defect, its location can suggest the presence of multiple defects within the floor of the oval fossa. Increased awareness of the defect should optimize its future clinical identification.

## 5. Conclusions

The vestibular defect is a true atrial septal defect located in the muscular antero-inferior rim of the oval fossa. The other type of true atrial septal defect occurs when there is a deficiency of the primary atrial septum or the flap valve at the floor of the oval fossa. Review of the episcopic data sets suggest that the defect exists because of failure, during embryonic development, of union of the components that bind the leading edge of the primary atrial septum to the atrioventricular junctions, either because of inadequate muscularisation or excessive apoptosis. The retrospective review of a large cohort of autopsied hearts suggests that the defect may be more common than previously thought. Increased awareness of the location of the defect should optimize its future clinical identification.

## Figures and Tables

**Table 1:** Characteristics of the specimens noted to have vestibular atrial septal defects.

Right-Sided Superior Caval Vein	68 (100)
Drainage of right-sided superior caval vein	
Right-sided atrium	68 (100)
Left-sided superior caval vein	4 (6)
Drainage of left-sided superior caval vein	
Coronary sinus	4 (100)
Pulmonary vein drainage	
Left-sided atrium	68 (100)
Right-sided atrium	
Normal	26 (38)
Dilated	42 (62)
Left-sided atrium	
Normal	46 (68)
Dilated	20 (29)
Hypoplastic	2 (3)
Oval fossa-type atrial septal defect	53 (78)
Coronary sinus defect	3 (4)
Common atrioventricular junction	10 (15)
Ventricular topology	
Right	67 (98)
Left	1 (2)
Atrioventricular connections	
Concordant	65 (94)
Discordant	1 (2)
Absent left-sided atrioventricular valve	1 (2)
Absent right-sided atrioventricular valve	1 (2)
Right-sided atrioventricular valve abnormality	40 (59)
Left-sided atrioventricular valve abnormality	19 (28)
Pulmonary atresia	17 (25)
Aortic atresia	1 (2)
Ventriculoarterial connections	
Concordant	60 (89)
Discordant	5 (7)
Double outlet right ventricle	3 (4)
Right-sided ventricle morphology	
Tripartite	51 (75)
Bipartite	12 (18)
Unipartite	5 (7)
Left-sided ventricle morphology	
Tripartite	66 (96)
Bipartite	1 (2)
Unipartite	1 (2)
Right outflow tract obstruction	
None	31 (46)
Infundibular	3 (5)
Valvar	6 (9)
Infundibular and valvar	11 (15)
Valvar atresia	17 (25)
Position of aorta relative to the pulmonary trunk	
Anterior	1 (2)
Anterior, rightward	7 (10)
Posterior, rightward	58 (84)
Side by side, rightward	1 (2)
Common arterial trunk	1 (2)
Left ventricular outflow tract obstruction	
None	54 (79)
Subvalvar	4 (6)
Valvar	7 (10)
Subvalvar and valvar	2 (3)
Valvar atresia	1 (2)
Interventricular communication	31 (46)
Aortic arch sidedness	
Left	66 (98)
Right	1 (2)
Coarctation of the aorta	4 (6)
Interruption of the aortic arch	2 (3)
Abnormal coronary artery origins or course	3 (6)
Arterial duct	
Absent	27 (40)
Present	33 (49)
Ligated	8 (11)
Cardiac apex	
Leftward	67 (98)
Pointing to middle	1 (2)
Trisomy 21	5 (7)
22 q deletion	1 (2)
